# Histopathological Findings in Laparoscopic Sleeve Gastrectomy Specimens from Patients with Obesity in Saudi Arabia

**DOI:** 10.1155/2018/1702705

**Published:** 2018-04-03

**Authors:** Khaldoon Aljerian

**Affiliations:** Forensic Medicine Unit, Department of Pathology, College of Medicine, King Saud University, Riyadh 12372, Saudi Arabia

## Abstract

Laparoscopic sleeve gastrectomy is a bariatric surgical procedure performed in patients with morbid obesity that provides the opportunity to review histopathological changes. The aim of this study was to characterize resected gastric specimens obtained from a Saudi patient population at a single center for a prospectively maintained database of laparoscopic sleeve gastrectomy cases. The histopathological findings from all patients undergoing laparoscopic sleeve gastrectomies at King Khalid University Hospital between 2010 and 2015 were retrospectively reviewed. Of the 602 cases reviewed, the majority (83.4% [*n* = 502]) exhibited chronic gastritis, whereas 22.3% (*n* = 134) involved *Helicobacter pylori* infections with active gastritis, 1% (*n* = 6) had intestinal metaplasia, and one case (0.17%) revealed gastric adenocarcinoma. As the findings revealed conditions that are treatable, I highly recommend histological examinations of all sleeve gastrectomy specimens from a Saudi patient population.

## 1. Introduction

Obesity and its various comorbidities currently comprise the most serious, most prevalent, and most challenging health concerns worldwide [1, 2]. For patients with severe obesity, high body mass indexes (BMIs), serious comorbidities, and extremely poor qualities of life, bariatric (weight loss) surgery can provide a substantial health benefit despite the operative risks [3]. Among the currently available bariatric surgical procedures and techniques, the most favored is laparoscopic sleeve gastrectomy (LSG) as either the sole treatment for obesity or as a component of complex bariatric surgical interventions [4]. The restrictive and metabolic effects of LSG result from the vertical resection of a large portion of the stomach, including the fundus [5–8]. These resection specimens are the sources for vast amounts of published data on histopathological changes in patients from developed countries and may provide a basis for monitoring long- and short-term effects of the operation. Thus, it is highly advised that bariatric/metabolic surgeons collect these specimens to provide further data on LSG.

Obesity and its comorbid conditions are widely prevalent in Saudi Arabia [9]. The World Health Organization reported that 23% and 36% of male and female Saudi individuals, respectively, over the age of 15 years were classified with obesity in 2010 [10]. The increasing incidence of obesity in this population has placed Saudi Arabia among the list of nations where bariatric surgery is becoming popular [11]. In this study, the histopathological findings in resected gastric specimens from patients undergoing LSG at a single center in Riyadh, Saudi Arabia, were examined for comparison with other published findings and to provide a basis for future guidelines for preoperative assessment and the design of more holistic treatment decisions.

## 2. Materials and Methods

This was a retrospective study conducted at the Department of Pathology, King Khalid University Hospital (KKUH), Saudi Arabia, after receiving approval from the institutional ethics committee. All patients undergoing LSGs for the treatment of morbid obesity between 2010 and 2015 were included in the study. Cases with partial gastrectomies for all other reasons were excluded.

Patient characteristics such as age, sex, and initial BMI were retrieved from clinical records. The findings of gross and histopathological examinations in the final biopsy reports were retrospectively reviewed in addition to the mentioned diagnosis. For a comprehensive analysis, the cases were categorized into the following groups on the basis of the biopsy report findings: (i) normal histology of stomach, (ii) chronic active gastritis with *Helicobacter pylori* infection, (iii) chronic gastritis alone, (iv) follicular lymphoid hyperplasia, (v) intestinal metaplasia, (vi) dysplasia/carcinoma in situ, (vii) carcinoma, and (viii) other pathological entities (such as polyps and leiomyoma). The data were entered and analyzed using the Statistical Package for Social Sciences (SPSS) version 20 for Windows. Simple descriptive statistics were applied for discrete variables such as sex and diagnosis. The means and standard errors and 95% confidence intervals (CIs) were calculated for continuous variables, such as age and BMI.

## 3. Results

Data from 602 patients were available for the retrospective analysis. The mean age of the patients was 31.36 ± 0.78 years (95% CI, 29.82–32.90 years), including 266 (44.2%) male and 336 (55.8%) female patients (male/female ratio, 4 : 5). Gross examinations of surgical specimens revealed that most (580/602 [96.4%]) had smooth, unremarkable outer surfaces, whereas congestion, hemorrhage, nodularity, flattening, sloughing, and edema were found in 60 (10%), 42 (7%), 4 (0.7%), 4 (0.7%), 2 (0.3%), and 2 (0.3%) specimens, respectively. The mean wall thickness was 5.76 ± 0.21 mm (95% CI, 5.34–6.18 mm).

**Figure 1:**
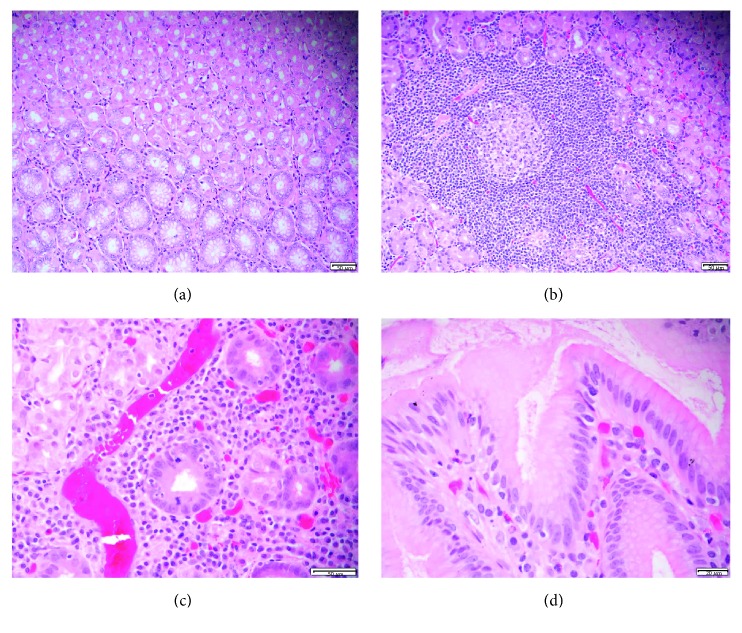
(a) Normal stomach mucosal epithelium and glands, paraffin embedded, H&E stained, ×200. (b) Chronic inflammation of stomach mucosal epithelium with mucosal follicle, paraffin embedded, H&E stained, ×200. (c) Acute and chronic inflammation of stomach mucosal epithelium with neutrophil infiltration of gland, paraffin embedded, H&E stained, ×400. (d) Stomach mucosal epithelium with *H. pylori*-like organisms on the surface, paraffin embedded, H&E stained, ×600.

Histological examinations indicated that the majority (83.4% [502/602]) of specimens had submucosal lymphocytic and plasmacytic infiltrates and thus constituted the diagnoses of chronic gastritis (Figure 1(b)). The second most frequent findings were the presence of *H. pylori* in the mucosal surfaces and crypts (22.3% [134/502]) and signs of active inflammation evidenced by the presence of neutrophils in the mucosal lining or the vicinity (22.3% [134/502]) for the diagnoses of active gastritis (Figures 1(c) and 1(d)). Most strikingly, 6 (1%) specimens were from patients with premalignant conditions, that is, intestinal metaplasia, and gastric adenocarcinoma was identified in 1 (0.17%) specimen (Table 1).

## 4. Discussion

As LSG is commonly used to treat obesity at KKUH, there is an opportunity to learn from the histopathological findings in the resected stomach specimens of these patients. There was a high prevalence of chronic gastritis (83.4%) and a moderate prevalence of active gastritis (22.3%), including cases with *H. pylori* infection. These findings are in line with those of Almazeedi et al. [12], who examined 656 LSG specimens from similarly aged Arab patients and showed that 74.4% had features of chronic gastritis, with 9.6% showing follicular gastritis and 1.8% with atrophic gastritis, but with only 7.3% of cases with *H. pylori* infections. They also reported four cases (0.6%) with gastric polyps, three cases (0.5%) with granulomatous disease, and one case (0.2%) each with a gastrointestinal stromal tumor, a gastrointestinal autonomic nerve tumor, intestinal metaplasia, collagenous gastritis, and crypt cell apoptosis [12]. In the present study, 1% of specimens had premalignant intestinal metaplasia, with one case of gastric adenocarcinoma. Similarly, a study of 27 patients with obesity undergoing LSG at another hospital in Saudi Arabia (King Fahd University Hospital, Al-Khobar) showed that the majority (74%) had chronic gastritis, with 11.1% showing signs of active gastritis associated with *H. pylori* infection [13]. However, the higher prevalence of *H. pylori* infection, in the present study, is in line with previous studies in Saudi Arabia [14–16].

A study of 33 Turkish patients with morbid obesity undergoing LSG by Gündoğan et al. [17] revealed interstitial lymphocytic infiltration (63.6%), hyperplasia of lymphoid follicles in the lamina propria (60.7%), and microvesiculation/dilatation of parietal cells (57.6%). A retrospective review of 48 LSG specimens from a U.S. hospital by Raess et al. [18] identified cases (8.4%) that warranted clinical follow-ups, including those with *H. pylori*-associated gastritis (observed in 5.2% of all cases and in 33.3% of cases of gastritis), autoimmune gastritis with microcarcinoid formation, necrotizing vasculitis, and intestinal metaplasia; neoplasms were identified at laparoscopy in 2 additional cases (0.8%). Another study of 125 U.S. patients undergoing LSG for obesity by Clapp [19] found that 49.7% of the resected specimens had histopathological findings, including acute (*n* = 4) and chronic (*n* = 61) gastritis, follicular lymphoid hyperplasia (*n* = 11), leiomyoma (*n* = 1), and fundic polyps (*n* = 2). Behrens et al. [20] followed up 34 Canadian patients (mean age, 48 years) with obesity undergoing LSG and found *H. pylori*-associated gastritis in only 2 (6%) patients and small gastrointestinal stromal tumors in 1 (3%) patient. A higher prevalence of *H. pylori* (33.3%) in association with chronic gastritis with inflammatory activity was reported in a Brazilian study by Onzi et al. [21].

In accordance with the high prevalence of gastritis among populations with obesity, the majority of histopathology results after LSG have an element of chronic gastritis. However, previous studies also show that at least a few cases undergoing LSG are found to harbor clinically significant pathologies that necessitate alterations in postoperative management [12]. A recent study by AbdullGaffar et al. [22] suggests that histological examination of LSG specimens from selected cases is more cost-effective than microscopic examination of all of them. However, the high prevalence of *H. pylori* infection and identification of premalignant and rare malignant cases by the present and other studies indicates that histological examinations of all specimens are warranted. This is particularly relevant for the Saudi population, for which life-threatening findings were noted that required an alteration of patient management.

## 5. Conclusions

I recommend routine histological examination of gastric specimens from patients after LSG for obesity across Saudi Arabia, considering the high prevalence of *H. pylori* infection in the population. We invite colleagues to add their experiences at various centers so that national guidelines may be formulated to address the preoperative and operative management of such patients and tackle these so-called “unanticipated findings” effectively.

## Figures and Tables

**Table 1:** Histological diagnoses identified by examining 602 patients.

Histological diagnosis	Percentage	Frequency
Chronic gastritis	83.4%	502
Active gastritis	22.3%	134
*H. pylori*	22.3%	134
Intestinal metaplasia	1%	6
Adenocarcinoma	0.17%	1
